# A Highly Hydrophobic and Flame-Retardant Melamine Sponge for Emergency Oil Spill Response

**DOI:** 10.3390/nano15241897

**Published:** 2025-12-17

**Authors:** Chengyong Zheng, Bo Wang, Wei Xie, Shuilai Qiu

**Affiliations:** 1 Western Pipeline Company of China Oil and Gas Pipeline Network Corporation, Urumqi 830011, China; 2 College of Safety and Ocean Engineering, China University of Petroleum-Beijing, 18 Fuxue Road, Beijing 102249, China

**Keywords:** oil-water separation, cyclic sorption, flame retardancy, oil spill remediation

## Abstract

Frequent crude oil spills during offshore oil and gas production and transportation have inflicted irreversible detrimental effects on both human activities and marine ecosystems; with particular risks of secondary disasters such as combustion and explosions. To address these challenges; advanced oil sorption technologies have been developed to overcome the inherent limitations of conventional remediation methods. In this study, a flame-retardant protective coating was fabricated on melamine sponge (MS) through precipitation polymerization of octa-aminopropyl polyhedral oligomeric silsesquioxane (POSS) and hexachlorocyclotriphosphazene (HCCP), endowing the MS@PPOS-PDMS-Si composite with exceptional char-forming capability. Secondary functional layer: By coupling the complementary physicochemical properties of polydimethylsiloxane (PDMS) and SiO_2_ nanofibers, we enabled them to function jointly, achieving superior performance in the material systems; this conferred enhanced hydrophobicity and structural stability to the MS matrix. Characterization results demonstrated a progressive reduction in peak heat release rate (PHRR) from 137.66 kW/m^2^ to118.35 kW/m^2^, 91.92 kW/m^2^, and ultimately 46.23 kW/m^2^, accompanied by a decrease in total smoke production (TSP) from 1.62 m^2^ to 0.76 m^2^, indicating significant smoke suppression. Furthermore, the water contact angle (WCA) exhibited substantial improvement from 0° (superhydrophilic) to 140.7° (highly hydrophobic). Cyclic sorption–desorption testing revealed maintained oil–water separation efficiency exceeding 95% after 10 operational cycles. These findings position the MS@PPOS-PDMS-Si composite as a promising candidate for emergency oil spill response and marine pollution remediation applications, demonstrating superior performance in fire safety, environmental durability, and operational reusability.

## 1. Introduction

Maritime oil transportation, a cornerstone of the global energy supply, is concomitantly a source of persistent ecological threats from accidental spills. These events lead to the pervasive release of toxic substances, which bioaccumulate and propagate through the food web, thereby endangering marine biodiversity and, ultimately, human health and food safety. This escalating urgency underscores the critical need for innovative remediation technologies, where nanotechnology-driven solutions and advanced materials hold significant promise for effective intervention [1]. Based on the principles of sustainable development and green chemistry, the demand for oil spill cleanup technologies has been increasing. Physical sorption [2], due to its simplicity, environmental friendliness, and recyclability, has been widely applied in various oil spill incidents [3]. However, traditional oil spill cleanup techniques such as skimming [4], oil booms, sedimentation, centrifugation, filtration [5], and biodegradation [6], suffer from low efficiency, time-consuming processes, and poor stability. Therefore, there is a pressing need for a cost-effective, environmentally friendly, and highly efficient oil–water separation material to overcome these technical limitations.

In the field of oil spill physical sorption, common adsorbent materials include aerogels [7,8,9,10], melamine sponges [11], polyurethane sponges, and biomass-based materials [12], which have garnered significant attention from researchers due to their eco-friendliness and low cost. These materials not only exhibit broad environmental adaptability but also enable efficient recovery of crude oil from marine environments. Melamine foam, in particular, has been selected as an oil-absorbing material owing to its ultra-lightweight porous structure, high open-cell ratio [13], excellent chemical stability, and reusability. Hexachlorocyclotriphosphazene (HCCP) possesses a molecular framework inherently rich in phosphorus and nitrogen [10], which facilitates a synergistic flame-retardant effect upon exposure to high heat or combustion [11]. The distinctive phosphorus–nitrogen architecture [12], coupled with its high reactivity, imparts the modified foam with superior high-temperature resistance to both deformation [13] and thermal degradation. This multifaceted performance improvement [14], mediated by a gas-phase and condensed-phase flame-retardant mechanism, offers a viable pathway for advanced applications requiring stringent flame retardancy and exceptional thermal stability [14]. Octaaminopropyl Polyhedral Oligomeric Silsesquioxane (POSS) is an organic–inorganic hybrid nanomaterial with a cage-like structure. Its rigid framework acts as a nanofiller uniformly dispersed within the foam matrix, hindering polymer chain movement through physical crosslinking points under high-temperature conditions, thereby improving rigidity. Polydimethylsiloxane (PDMS), synthesized from polydimethylsilane as a precursor [15], offers advantages such as thermal stability [16], hydrophobicity, and excellent resilience [17]. Meanwhile, SiO_2_ nanofibers possess superior flexibility, which helps preserve the structural integrity of the foam skeleton [18] while also increasing surface roughness [19] to enhance hydrophobic properties. However, while POSS improves thermal stability, its flame-retardant efficiency remains insufficient. Using POSS alone for material modification can lead to brittleness, hindering practical recyclability. Similarly, HCCP, despite its outstanding flame retardancy, induces material embrittlement [20] and releases toxic gases such as hydrogen chloride and chlorinated aromatics under high-temperature combustion, causing air pollution. Although crosslinked PDMS and SiO_2_ nanofibers can enhance mechanical strength and hydrophobicity [21], they significantly reduce porosity [22].To address the drawbacks of conventional melamine foam—such as high brittleness, poor resilience, inadequate heat resistance, weak hydrophobicity/oleophilicity [23], and susceptibility to environmental influences, this study integrates the advantages of the aforementioned materials by modifying melamine foam with POSS-containing phosphazene polymers, crosslinked PDMS, and SiO_2_ nanofibers.

This study innovatively combines octaaminopropyl POSS, hexachlorocyclotriphosphazene (HCCP), and silica (SiO_2_) nanofibers to successfully overcome the critical limitations associated with single-component modifications. Through a condensation reaction between HCCP and octaaminopropyl POSS, a novel POSS-containing phosphazene polymer was synthesized. This creates a phosphorus–nitrogen–silicon ternary synergistic system that forms a more stable phosphosiloxane crosslinked network, significantly enhancing char layer strength and thermal insulation performance. The chlorine atoms in HCCP react with the amino groups of POSS to form phosphazene-POSS crosslinking points, which improve matrix integrity while reducing the risk of chlorine residue. Subsequently, incorporating silica (SiO_2_) nanofibers into the HCCP-POSS hybrid-modified foam further enhances the composite’s overall performance through synergistic effects. The anchoring of SiO_2_ nanofibers on the foam skeleton [24] effectively reduces the surface energy of the foam and increases the water contact angle, achieving superior hydrophobicity and oleophilicity. The multi-component synergistic strategy successfully integrates superior flame retardancy, thermal stability, mechanical robustness, and environmental safety. This represents a significant step toward the practical deployment of the developed material as a high-performance nano-adsorbent for oil spill cleanup.

## 2. Experimental Section

### 2.1. Materials

Melamine foam was purchased from Chengdu Rongyulong Technology Co., Ltd. Octaaminophenyl polyhedral oligomeric silsesquioxane (POSS) was obtained from Hubei Meddox Biotechnology Co., Ltd., Sichuan, China. Hexachlorocyclotriphosphazene (HCCP, purity ≥ 98%), triethylamine (TEA, purity ≥ 99%), oxalic acid, and tetraethyl orthosilicate (TEOS, purity ≥ 98%) were acquired from Shanghai Aladdin Biochemical Technology Co., Ltd., Shanghai, China. Polydimethylsiloxane (PDMS) and the corresponding curing agent (weight ratio 10:1) were supplied by Dow Corning Corporation, USA. The following solvents were purchased from Shanghai Aladdin Biochemical Technology Co., Ltd.: carbon tetrachloride, chloroform, dichloromethane, dichloroethane, tetrahydrofuran (THF), acetone, ethyl acetate, N-methyl-2-pyrrolidone (NMP), cyclohexane, dimethylformamide (DMF), and heptane. Petroleum ether and oil for softening leather were obtained from Jinan Weizhen Chemical Co., Ltd., Beijing, China. Deionized water, used in all experiments, was produced using an ultrapure water system purchased from Anhui Labrary Instrument Technology Co., Ltd., Anhui, China.

### 2.2. Preparation of SiO_2_ Nanofibers

The spinnable silica sol precursor solution was first prepared by mixing tetraethyl orthosilicate (TEOS), absolute ethanol, deionized water, and oxalic acid (C_2_H_2_O_4_) at a weight ratio of 1:0.8:0.3:0.007, followed by vigorous stirring for 8 h at room temperature. To homogenize the spinning solution, 0.12 g of polyethylene oxide (PEO) was added as a sacrificial polymer template to 10 g of the silica sol, with subsequent stirring for 4 h at ambient temperature. The hybrid nanofiber membrane was then fabricated using an electrospinning apparatus with the following parameters: applied voltage of 15 kV, constant infusion rate of 2 mL/h, needle-to-collector distance of 20 cm, and an injection needle with an inner diameter of 0.7 mm. Finally, the obtained hybrid nanofibers were calcined in air at 800 °C for 1 h in a muffle furnace with a heating rate of 5 °C/min to ensure complete removal of the PEO template from the precursor nanofibers, yielding pure SiO_2_ nanofibers.

### 2.3. Fabrication of MS@PPOS-PDMS-Si

**Figure 1:**
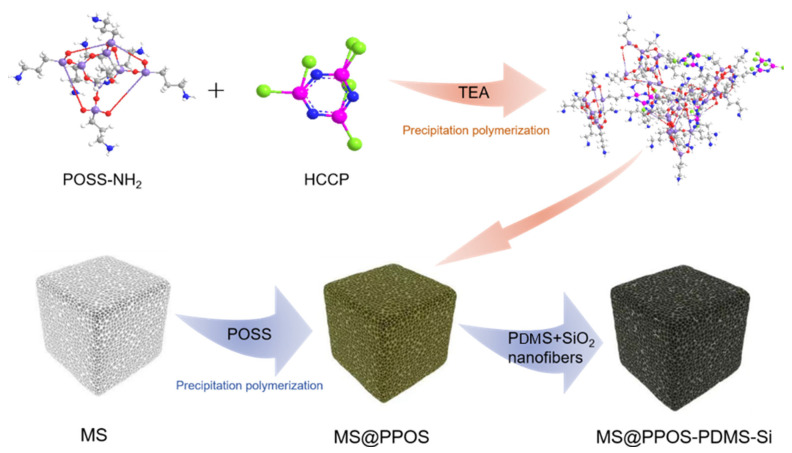
Schematic illustration of the fabrication process for MS@PPOS-PDMS-Si composite.

Initially, octaaminopropyl polyhedral oligomeric silsesquioxane (POSS) was dissolved in tetrahydrofuran (THF), followed by the addition of 0.3 g triethylamine (TEA) as a catalyst under ultrasonication until complete dissolution. Hexachlorocyclotriphosphazene (HCCP) was separately dissolved in THF to form a transparent solution. Under ice-bath conditions, the HCCP solution was slowly added to the POSS/TEA mixture to initiate a polycondensation reaction, yielding a POSS-containing phosphazene polymer hybrid solution. During the precipitation polymerization reaction, the phosphazene polymer is made to grow in situ on the surface of MS, and the loading of the phosphazene polymer on the surface of MS is controlled by the concentration of the reaction monomer. Subsequent washing with ethanol and deionized water removed residual particles, followed by drying at 60 °C for 3 h to obtain the flame-retardant sponge (denoted as MS@PPOS). This pretreatment enabled in situ growth of the phosphazene polymer on the MS surface. For PDMS/SiO_2_ modification, crosslinkable PDMS (base-to-curing agent mass ratio = 10:1) was mixed with anhydrous ethanol (10:1 mass ratio) under stirring to form Solution A. An certain amount of SiO_2_ nanofibers was then dispersed in Solution A via ultrasonication to prepare Solution B. The flame-retardant MS was impregnated with Solution B and cured at 60 °C, yielding the final composite MS@PPOS-PDMS-Si. In this modification process, the SiO_2_ nanofibers functionally modified the surface of the PDMS-treated flame-retardant MS, while the curing agent facilitated surface crosslinking of the flame-retardant matrix. The incorporation of PDMS endowed the flame-retardant MS with excellent hydrophobicity, whereas the SiO_2_ nanofibers not only enhanced surface roughness, but also improved mechanical flexibility through their reinforcing effect. The collective action of these modification strategies, through their integrated effect, ultimately led to the successful fabrication of the high-performance MS@PPOS-PDMS-Si composite. The complete fabrication procedure is schematically illustrated in Figure 1.

### 2.4. Wettability Characterization

The water contact angle (WCA), serving as a critical parameter for evaluating the surface wettability of sponge materials, quantitatively reflects the hydrophobic/hydrophilic characteristics [25]. The prepared foam sample was placed on the testing platform, and a microsyringe was used to precisely dispense 5 μL of deionized water vertically onto its surface. The optical system was carefully adjusted, including optimization of the light source intensity and camera focus, to obtain high-contrast droplet images with well-defined boundaries between the solid–liquid–vapor phases. Subsequently, the static water contact angles at multiple sample locations were measured through combined image analysis and tangent methods, with the final reported value representing the arithmetic mean of these measurements.

### 2.5. Sorption Performance Test

The sorption performance [26] was evaluated by immersing modified samples (2 cm × 2 cm × 1 cm) in various oils and organic solvents for predetermined durations to determine their absorption capacity. The absorption capacity (Q, g/g) of the composite sponge for different oils and organic solvents was calculated according to Equation (1):

(1) $$Q=\frac{m_{1}-m_{0}}{m_{0}}$$

where m_0_ and m_1_ represent the mass (g) of the composite sponge before and after saturation with the test liquid, respectively.

### 2.6. Oil–Water Separation Efficiency Test

The oil–water separation efficiency (η, %) [27] was quantitatively evaluated using a customized separation apparatus containing the modified sample (2 cm × 2 cm × 1 cm). The separation performance was determined by measuring the oil content in the oil–water emulsion before and after the separation process. The separation efficiency was calculated according to Equation (2):

(2) $$\eta =\frac{M_{1}-M_{0}}{M_{1}}\times 100$$

where M_0_ and M_1_ (g) represent the mass of oil in the oil–water emulsion before and after separation, respectively.

### 2.7. Cone Calorimeter Test

The cone calorimetry test (Cone) was conducted on the cone calorimeter (Suzhou TESTech Testing Instrument Technology Co., Ltd.) according to the ISO 5660 standard. The sample size was 100 × 100 × 3 mm^3^. During the test, the sample was wrapped with aluminum foil paper, and the heat radiation flux was 35 kW/m^2^.

## 3. Results and Discussion

### 3.1. Structural and Morphological Analysis

**Figure 2:**
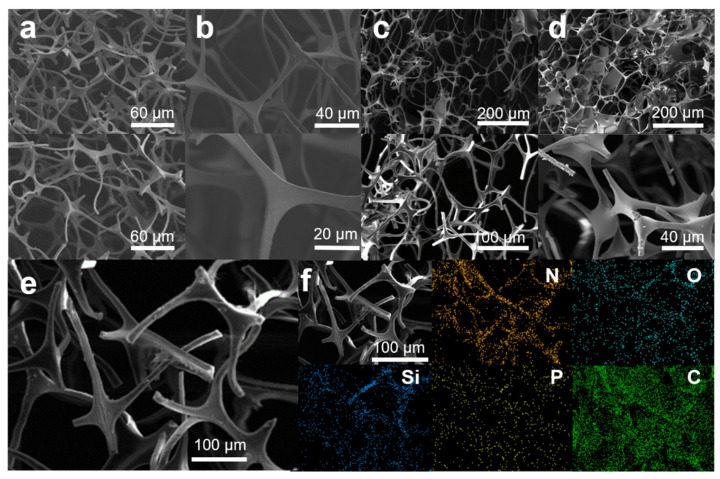
SEM images of (**a**) MS, (**b**) MS@PPOS, (**c**) MS@PPOS-PDMS, and (**d**) MS@PPOS-PDMS-Si; (**e**) SEM image and (**f**) EDS mapping of MS@PPOS-PDMS-Si.

The microstructural characteristics and surface composition of MS, MS@PPOS, MS@PPOS-PDMS, and MS@PPOS-PDMS-Si were systematically investigated through scanning electron microscopy with energy-dispersive X-ray spectroscopy (SEM-EDS), as illustrated in Figure 2. As shown in Figure 2a, the pristine melamine sponge (MS) exhibits a smooth internal architecture with uniform pore structure. Following modification with POSS-containing phosphazene polymer (Figure 2b), distinct surface texturing emerges, confirming the successful in situ growth of octaaminopropyl POSS-phosphazene polymer on the MS framework. Figure 2c,d reveal a substantial conformal coating on the MS struts, accompanied by significantly enhanced surface roughness, demonstrating the effective anchoring of both the highly hydrophobic PDMS layer and SiO_2_ nanofibers onto the sponge matrix. EDS analysis of MS@PPOS-PDMS-Si (Figure 2f) confirms its chemical composition evolution. While the original melamine sponge contains only C and N elements, the modified composite shows homogeneous distribution of Si, O, and P throughout its framework, providing definitive evidence for the successful incorporation of POSS-phosphazene polymer, highly hydrophobic PDMS coating, and SiO_2_ nanofibers. Notably, abundant micro/nano-composite structures were successfully engineered on the material surface via the modification process (Figure 2c,d), serving as stable air-entrapment pockets in the foam skeleton. This hierarchical architecture facilitates a pronounced air-cushion effect that drastically reduces the solid–liquid contact area, which is fundamentally responsible for the highly hydrophobic performance observed in the MS@PPOS-PDMS-Si sample.

### 3.2. Oil–Water Separation Performance

**Figure 3:**
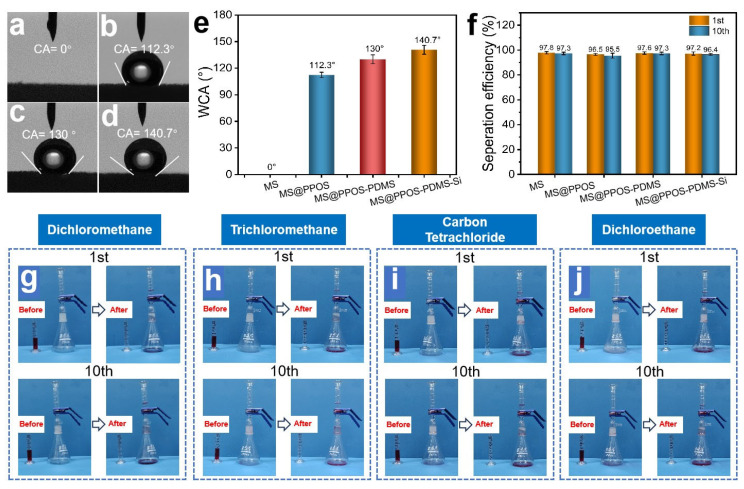
Optical images of water contact angles on (**a**) MS, (**b**) MS@PPOS, (**c**) MS@PPOS-PDMS, and (**d**) MS@PPOS-PDMS-Si surfaces. (**e**) Comparative water contact angle measurements of the four samples. (**f**) Oil–water separation efficiency during the 1st and 10th sorption-squeezing cycles for all samples. Oil–water separation performance of MS@PPOS-PDMS-Si for (**g**) dichloromethane, (**h**) chloroform, (**i**) carbon tetrachloride, and (**j**) dichloroethane during the 1st and 10th cycles.

The oil–water separation capability [28] represents the most critical functionality of flame-retardant hydrophobic MSs for practical oil spill remediation applications, enabling simultaneous oil–water separation and oil recovery [13]. As illustrated in Figure 3a, the pristine MS exhibited complete hydrophilicity with a water contact angle (WCA) of 0°. After surface modification, MS@PPOS, MS@PPOS-PDMS, and MS@PPOS-PDMS-Si demonstrated significantly enhanced hydrophobicity with WCAs of 112.3°, 130°, and 140.7°, respectively (Figure 3b–d). This remarkable hydrophobic transformation originated from two key factors: (1) the PDMS coating effectively passivated hydrophilic groups on the foam skeleton, and (2) the abundant methyl and siloxane groups in both PDMS and SiO_2_ dramatically reduced the surface energy of the melamine matrix. To quantitatively evaluate the separation performance, systematic oil–water separation tests were conducted using MS, MS@PPOS, MS@PPOS-PDMS, and MS@PPOS-PDMS-Si. As shown in Figure 3f, all modified materials maintained high separation efficiencies with initial values of 97.8%, 96.5%, 97.6%, and 97.2% for the four samples, respectively. After 10 consecutive separation cycles, the efficiencies remained at 97.3%, 95.5%, 97.3%, and 96.4%, demonstrating negligible performance degradation [29]. This significant hydrophobicity enhancement originates from the combined action of the PDMS coating, which suppresses the hydrophilic groups on the foam skeleton, and the abundant methyl/siloxane functionalities from PDMS and SiO_2_, which markedly lower the surface energy of the melamine matrix. To further evaluate the oil–water separation performance of MS@PPOS-PDMS-Si, gravity-driven oil–water separation experiments were conducted to assess its separation efficiency. As shown in Figure 3g–j, the first and tenth separation cycles of MS@PPOS-PDMS-Si were performed for oil–water systems composed of dichloromethane, chloroform, carbon tetrachloride, and dichloroethane with water, respectively. The experimental results demonstrate that in both the first and tenth cycles, the aqueous phase was effectively retained above the MS@PPOS-PDMS-Si layer, while the four organic solvents rapidly permeated through the material and accumulated at the bottom of the container, achieving highly efficient oil–water separation.

### 3.3. Sorption Performance and Cyclic Stability

**Figure 4:**
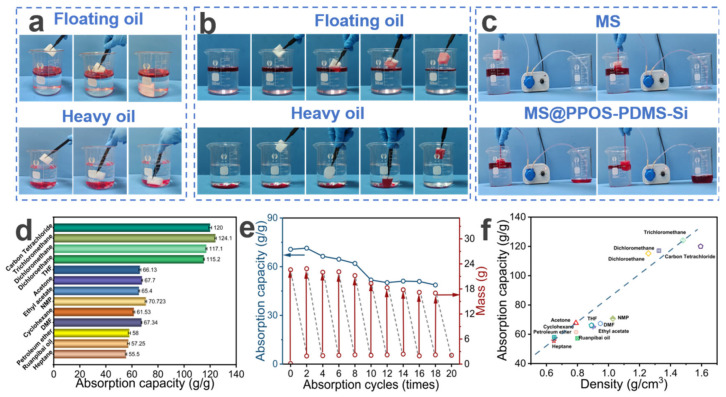
(**a**) MS and (**b**) MS@PPOS-PDMS-Si demonstrating sorption of light/heavy oils (stained with Oil Red O). (**c**) Comparative oil–water separation using a peristaltic pump system. (**d**) Absorption capacities for various oils and organic solvents. (**e**) Cyclic sorption performance over 20 cycles. (**f**) Correlation between absorption capacity and density of different oils/solvents.

MS exhibits certain oil–water sorption capabilities but lacks selective sorption. As shown in Figure 4a, unmodified MS demonstrates non-selective sorption toward mixed solutions of light and heavy oils, leading to significant water uptake and subsequent sinking, thereby failing to achieve effective oil–water separation. In contrast, MS@PPOS-PDMS-Si effectively overcomes this limitation. As illustrated in Figure 4b, owing to its excellent hydrophobicity, MS@PPOS-PDMS-Si achieves selective sorption of both light and heavy oils, significantly improving its oil absorption rate. To further validate the oil–water separation performance and petroleum ether recovery efficiency of MS and MS@PPOS-PDMS-Si, a peristaltic pump was employed to compare their separation efficiencies. As shown in Figure 4c, the oil–water separation performance of MS is significantly inferior to that of MS@PPOS-PDMS-Si, with the right beaker clearly containing substantial amounts of both water and oil. This comparative experiment conclusively demonstrates the superior selective oil sorption and separation efficiency of MS@PPOS-PDMS-Si, providing critical experimental evidence for its application in oil spill emergency response. Additionally, MS@PPOS-PDMS-Si exhibits excellent sorption capacity for various organic solvents. As depicted in Figure 4d, the maximum absorption capacity of MS@PPOS-PDMS-Si for different organic solvents (carbon tetrachloride, chloroform, dichloromethane, dichloroethane, THF, acetone, ethyl acetate, NMP, cyclohexane, DMF, petroleum ether, oil for softening leather, and heptane) reaches 124.1 g/g, with a minimum absorption capacity of 55.5 g/g. The absorption range for other solvents falls between 57.25 and 120 g/g. The sorption capacity of MS@PPOS-PDMS-Si for various organic solvents and oils is correlated with their densities. As shown in Figure 4f, the sorption capacity increases with solvent density, exhibiting an approximately linear relationship. For instance, since chloroform has a higher density than dichloroethane, MS@PPOS-PDMS-Si shows a higher sorption capacity for chloroform. This suggests that higher oil density leads to better absorption performance in MS@PPOS-PDMS-Si. MS@PPOS-PDMS-Si exhibits superior sorption capacity for heavy oils compared to light oils. The material’s density-governed sorption behavior is clearly illustrated in Figure 4b, where its superior uptake of heavy oils over light oils consistently follows the previously identified trend of increasing capacity with solvent density. In practical applications, the reusability of materials can substantially reduce engineering costs while enhancing sustainability and market viability, providing critical support for their large-scale application in oil spill emergency response. To evaluate the cyclic stability and durability of MS@PPOS-PDMS-Si, a 20-cycle sorption-squeezing–drying test was conducted. As illustrated in Figure 4e, after 18 cycles, the absorption capacity of MS@PPOS-PDMS-Si for diesel decreased from an initial 70.7 g/g to 48.7 g/g, while still maintaining excellent sorption performance. This outstanding cyclic stability can be attributed to the incorporation of SiO_2_ nanofibers in MS@PPOS-PDMS-Si. Their exceptional flexibility and mechanical strength effectively mitigate fatigue-induced structural damage during repeated cycles, thereby preserving the porous structure and sorption performance of the material. Furthermore, these results confirm the successful coating and stable anchoring of SiO_2_ nanofibers onto the MS framework.

### 3.4. Flame Retardancy Performance

**Figure 5:**
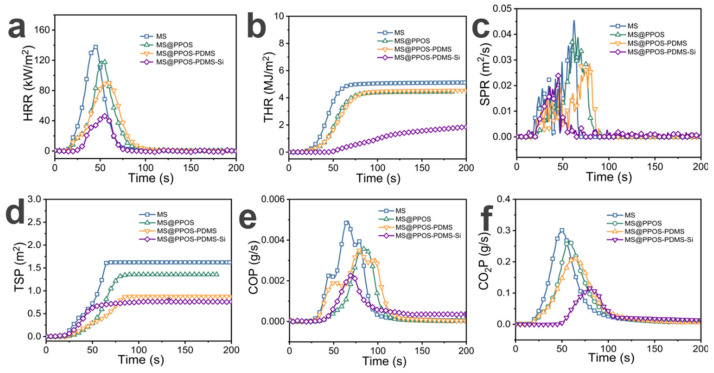
(**a**) Heat release rate (HRR). (**b**) Total heat release (THR). (**c**) Smoke production rate (SPR). (**d**) Total smoke production (TSP). (**e**) CO production rate (COP). (**f**) CO_2_ production rate (CO_2_P).

In marine oil spill emergency response [30], oil-absorbing sponges [31] are widely used due to their high porosity and lightweight characteristics. However, the high oxygen [32] permeability and flammability resulting from this high porosity [33] make them susceptible to oil-induced fires under intense marine radiation conditions, posing significant safety hazards in practical applications. To quantitatively evaluate the flame retardancy of MS@PPOS-PDMS-Si, cone calorimetry tests were conducted on different MS composite components, including heat release rate (HRR), total heat release (THR), smoke production rate (SPR), total smoke production (TSP), as well as carbon monoxide (CO) and carbon dioxide (CO_2_) production rates. As shown in Figure 5a, the peak HRR of pristine MS reached 139.44 kW/m^2^. With the incorporation of octaaminopropyl POSS, PDMS, and SiO_2_, the peak HRR values of modified MS gradually decreased to 119.66 kW/m^2^, 91.92 kW/m^2^, and 46.72 kW/m^2^, respectively. Notably, MS@PPOS-PDMS-Si exhibited a 66.49% reduction in peak HRR compared to pristine MS, demonstrating that octaaminopropyl POSS, PDMS, and SiO_2_ nanofibers effectively suppress the heat release of MS. Figure 5b presents the THR results, where MS, MS@PPOS, MS@PPOS-PDMS, and MS@PPOS-PDMS-Si showed maximum THR values of 5.12 MJ/m^2^, 4.44 MJ/m^2^, 4.53 MJ/m^2^, and 1.85 MJ/m^2^, respectively. These represent reductions of 13.28%, 11.52%, and 63.87% compared to pristine MS. This significant improvement is attributed to the condensation and crosslinking reactions between Si-OH groups on the PDMS and SiO_2_ layers under high temperatures, forming a protective Si-O-Si barrier that effectively isolates oxygen and achieves flame retardancy. Smoke and toxic gas emissions can cause irreversible harm to human health. As illustrated in Figure 5c, MS@PPOS-PDMS-Si exhibited a peak SPR of 0.02 m^2^/s, representing a 44.4% reduction compared to pristine MS. From the perspective of TSP (Figure 5d), the maximum TSP value of pristine MS was 1.62 m^2^, while MS@PPOS, MS@PPOS-PDMS, and MS@PPOS-PDMS-Si showed peak TSP values of 1.36 m^2^, 0.88 m^2^, and 0.76 m^2^, respectively. This suppression of smoke generation during combustion, achieved through the incorporation of octaaminopropyl POSS, PDMS, and SiO_2_, provides critical time for personnel evacuation. The reduction in toxic gas emissions further confirms the excellent flame retardancy of MS@PPOS-PDMS-Si. Figure 5e,f present the CO and CO_2_ production rates, respectively. The peak CO production rate decreased from 0.004 g/s to 0.002 g/s, while the maximum CO_2_ production rate progressively reduced from 0.30 g/s to 0.25 g/s, 0.21 g/s, and 0.12 g/s with successive material modifications, demonstrating enhanced suppression of hazardous gas emissions.

### 3.5. Char Residue Analysis

**Figure 6:**
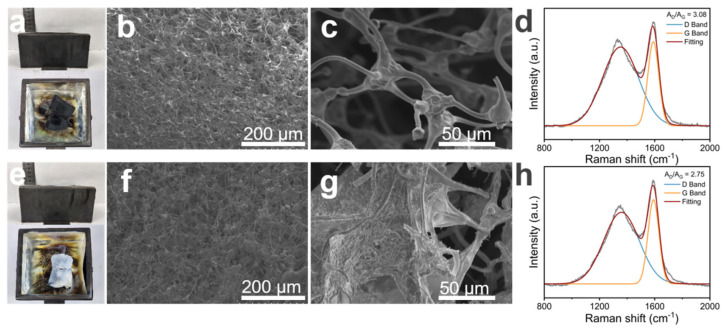
Post−combustion characterization: MS residue analysis showing (**a**) macroscopic morphology, (**b**) SEM micrograph, (**c**) Raman spectrum, and (**d**) corresponding spectral interpretation; MS@PPOS-PDMS-Si residue analysis presenting (**e**) macroscopic morphology, (**f**) SEM micrograph, (**g**) Raman spectrum, and (**h**) spectral deconvolution.

To further investigate the flame-retardant mechanism of MS@PPOS-PDMS-Si, structural characterization and Raman spectroscopy were performed on its residual char layer. As shown in Figure 6a,e, pristine MS underwent significant shrinkage after high-temperature combustion, whereas MS@PPOS-PDMS-Si exhibited only minor shrinkage at the edges. Scanning electron microscopy (SEM) provided clearer insights into their structural changes post-combustion. A comparison of Figure 6b,f revealed that during carbonization, octaaminopropyl POSS, PDMS, and SiO_2_ established a synergistic phosphorus–nitrogen–silicon system, which enhanced the crosslinking density and thermal stability of the char residue. This confirms that phosphorus (P) and silicon (Si) are key elements contributing to the flame-retardant properties of the composite sponge. In Figure 6c, the MS framework displayed fractures, highlighting its poor mechanical stability. In contrast, Figure 6g demonstrated that the incorporation of PDMS and SiO_2_ maintained structural integrity, thereby enhancing the cyclic sorption capacity of MS. Raman spectroscopy provided quantitative evidence of the composite sponge’s flame retardancy. As illustrated in Figure 6d,h, the A_D_/A_G_ ratios of MS and MS@PPOS-PDMS-Si were 3.08 and 2.75, respectively. The lower ratio for MS@PPOS-PDMS-Si indicates higher graphitization degree of the char layer. During combustion, this dense char layer acted as a barrier, shielding oxygen and heat transfer, thereby improving the thermal–oxidative stability of the composite sponge.

## 4. Conclusions

MS@PPOS-PDMS-Si possesses excellent hydrophobicity, mechanical properties, and flame retardancy. In this study, the melamine sponge was modified by anchoring the phosphorus–nitrogen-containing polymer, which was generated from the reaction between octaaminopropyl POSS and hexachlorocyclotriphosphazene, onto the surface of the melamine sponge. With the addition of octaaminopropyl POSS, hexachlorocyclotriphosphazene, and SiO_2_, the maximum value of the heat release rate of the modified MS gradually decreased from 139.44 kW/m^2^ to 119.66 kW/m^2^, 91.92 kW/m^2^, and 46.72 kW/m^2^. The total amount of smoke released also changed from the initial 1.62 m^2^ to 0.76 m^2^. The peak value of the CO generation rate decreased from 0.004 g/s to 0.002 g/s, and the maximum value of the CO_2_ generation rate decreased from 0.30 g/s to 0.12 g/s. The release amounts of flue gas and harmful gases were significantly reduced. The A_D_/A_G_ values of MS and MS@PPOS-PDMS-Si are 3.08 and 2.75, respectively, indicating that the thermal stability of the modified MS has been significantly improved compared with that of the original MS. In terms of sorption, its absorption capacity for various organic solvents and oils remains stable within the range of 55.5–124.1 g/g. To achieve efficient oil–water separation and recovery, the combined contribution of PDMS polymers and SiO_2_ led to a significant improvement in both the surface roughness and framework flexibility of the MS, successfully increasing the water contact angle from 0° to 140.7°. Moreover, after 10 cycles of sorption, the oil–water separation efficiency is still maintained above 95%. The collective enhancements in flame retardancy, sorption capacity, and cyclic stability position this material as a promising solution to key challenges of conventional sorbents, such as easy ignition, low efficiency, and short service life in practical scenarios.

## Data Availability

The raw data supporting the conclusions of this article will be made available by the authors on request.
